# Modelling and extraction of variability in free-text medication prescriptions from an anonymised primary care electronic medical record research database

**DOI:** 10.1186/s12911-016-0255-x

**Published:** 2016-02-09

**Authors:** George Karystianis, Therese Sheppard, William G. Dixon, Goran Nenadic

**Affiliations:** 1 School of Computer Science, University of Manchester, Manchester, UK; 2 The Christie NHS Foundation Trust, Manchester, UK; 3 Arthritis Research UK Centre for Epidemiology, University of Manchester, Manchester, UK; 4 The Farr Institute of Health Informatics Research, Health eResearch Centre, Manchester, UK; 5 Manchester Institute of Biotechnology, University of Manchester, Manchester, UK

**Keywords:** Text mining, Natural language processing, Dose information, Prescriptions, CPRD

## Abstract

**Background:**

Free-text medication prescriptions contain detailed instruction information that is key when preparing drug data for analysis. The objective of this study was to develop a novel model and automated text-mining method to extract detailed structured medication information from free-text prescriptions and explore their variability (e.g. optional dosages) in primary care research databases.

**Methods:**

We introduce a prescription model that provides minimum and maximum values for dose number, frequency and interval, allowing modelling variability and flexibility within a drug prescription. We developed a text mining system that relies on rules to extract such structured information from prescription free-text dosage instructions. The system was applied to medication prescriptions from an anonymised primary care electronic record database (Clinical Practice Research Datalink, CPRD).

**Results:**

We have evaluated our approach on a test set of 220 CPRD prescription free-text directions. The system achieved an overall accuracy of 91 % at the prescription level, with 97 % accuracy across the attribute levels. We then further analysed over 56,000 most common free text prescriptions from CPRD records and found that 1 in 4 has inherent variability, i.e. a choice in taking medication specified by different minimum and maximum doses, duration or frequency.

**Conclusions:**

Our approach provides an accurate, automated way of coding prescription free text information, including information about flexibility and variability within a prescription. The method allows the researcher to decide how best to prepare the prescription data for drug efficacy and safety analyses in any given setting, and test various scenarios and their impact.

**Electronic supplementary material:**

The online version of this article (doi:10.1186/s12911-016-0255-x) contains supplementary material, which is available to authorized users.

## Background

Electronic health records (EHRs) are becoming widely adopted in national healthcare systems in both primary and secondary care. By collecting and aggregating data from anonymised EHRs, research databases have been established to support large-scale epidemiological analysis: for example, the Clinical Practice Research Datalink (CPRD, http://www.cprd.com/) provides comprehensive longitudinal primary care data for over 11 million UK patients to support observational research, making it the world’s largest computerized healthcare database [1]. Examples of research supported by CPRD include drug utilisation studies [2], pharmacoepidemiology [3] and health services research [4]. Analysis of population data within such databases is often dependent on coded or structured information. For example, data on medication prescriptions include structured and coded information about variables like date of prescription, medication type, dosage and number of tablets. However, large parts of EHRs are un- or semi-structured. During medication prescribing, the doctor is able to provide free text directions to the patient indicating some details that are not coded (e.g. an option to take a tablet when needed up to a maximum number of times a day). In order to utilise this information in large population analysis, additional processing to extract structured information is required. In many cases, manual efforts have been undertaken to identify and extract key information, but such approaches are extremely time consuming and often inconsistent and incomplete [5–7]. In this manuscript we present an automated methodology to extract and represent prescription instruction information in a structured form, capturing, in particular, the variability and flexibility of dosage information. For example, instruction ‘two tablets up to three times a day’, could mean 0, 2, 4 or 6 tablets and we here propose an approach to model, identify and record flexibility in drug directions as prescribed by doctors. Our main motivation is to support researchers in making transparent decisions when preparing prescription data for further processing.

To identify key clinical information from unstructured and semi-structured text, automated text mining has been used for over 30 years [8–11]. It relies on various lexical, syntactic and semantic techniques in order to recognise and link various concepts [12–14]. Methods have been applied to discharge summaries, clinical notes, reports, health records and journal articles [15–18], and the results – despite numerous challenges – have demonstrated the potential of text mining to streamline data collection, improve healthcare, decrease health costs, reduce the risk of medical errors and enhance medical understanding [18–21].

There have been a number of studies that specifically focused on the extraction of medications from clinical text [10, 22, 23]. These have mostly focused on the identification of textual expressions that refer to drug usage, extracting characteristics such as medication dose (e.g., “*2 tablets”, “5 ml”*), mode of administration (e.g., “*orally”*), frequency (e.g., “*every two hours”*) and duration (e.g., “*for 2 days”*). However, the aim was often to identify mentions of these characteristics in text, rather than to convert them into structured values that can be used directly for data analytics or epidemiological research. This is in particular important in cases where prescriptions have an optional dose (e.g., “*2-3 tablets”*), optional frequency (e.g., “*twice a day as required”*) or an interval gap between the medication administration (e.g., “*every other day”*). In these cases, existing text mining approaches do not aim to provide full information in a structured form (e.g., that a tablet is to be taken either 2 or 3 times), but rather extract *textual spans* that need further processing in order to identify the options. For example, from *“2-4 tablets each morning as needed”*, existing approaches would extract “*2-4 tablets*” as dose number, without specifying that the patient has an option to take between 2 (minimum dose number) and 4 (maximum dose number), or even to skip it completely (“*as needed”*). Having this detailed information explicitly represented and extracted is key for allowing healthcare data analysts to study the impact of different prescription options and plans. For example, “*take 1-2 tablets per day*” can be used to analyse the effects of using a minimum dose of 1 or a maximum dose of 2 tablets, rather than assuming that the patient will take an average of 1.5 tablets each day.

This paper presents a novel approach to model and extract structured detailed values that represent the prescribed drug usage to support subsequent epidemiological studies. Specifically, we introduce a model to represent the variability and flexibility in drug directions, including minimum and maximum values for drug dosage, frequency and interval of administration, as well as optional choices. We also present a text mining system that enables the identification and structuring of the model components, and evaluate its performance on a subset of the CPRD prescription records. Using the system, we then provide descriptive statistics of the medication information variability having run the tool on a set of 56,000 most common free text instructions from the CPRD database.

### Related work

There are several approaches to the extraction of medication information from unstructured clinical notes. One of the early examples is that of Evans and colleagues [5], who introduced a rule-based approach that identified targeted dose information (dose level and frequency, duration, rate, necessity, purpose, quantity) from discharge summaries with a relatively good performance (an accuracy of 80 %). More recently, the identification of medication mentions and corresponding attributes (dose, mode of administration, frequency, duration and reason for administration) in hospital discharge summaries was a focus of the 2009 i2b2 Clinical Data Challenge, which attracted 19 international teams with different methodologies ranging from rule-based systems to machine learning [24]. A number of systems (e.g. [10, 23, 25]) utilized rule-based methods and reported F-scores (the harmonic mean of precision (i.e. positive predictive value) and recall (i.e. sensitivity)) between 78 % and 86 %. Patrick et al. [9], on the other hand, relied on machine learning techniques (conditional random fields (CRFs) and support vector machines (SVMs)) with an overall 86 % F-score. Following the challenge, Doan et al. [26] applied various classifiers with different voting strategies in order to combine outputs from three individual classifiers (a rule-based system, SVM and CRF) with an F-score of 91 %. More recently, Sohn et al. [27] described a medication extraction and normalization system (MedXN) that is based on the RxNorm dictionary combined with inference rules. They aimed to identify various components of medication prescriptions from clinical notes including drug dosage and frequency with a 92 % and 84 % F-scores respectively. Finally, MacKinlay et al. [28] proposed a dependency graph-based system for the recognition of dosage information such as the minimum and maximum dose numbers and the dose unit with an overall accuracy of 80 %, optional doses with 88 % accuracy and the dose frequency with 90 % accuracy. However, they have not focused on the identification of the minimum and maximum dose frequency, nor the minimum and maximum dose interval.

## Methods

### Prescription data model

The CPRD database contains over 56,000 most common free-text prescription rubrics that GPs have used to instruct administration of medications. Examples include:

**Table:** 

*one every 8 hrs* *5mls daily when required* *two drops every 3 hrs when required* *apply sparingly 1-2 times daily* *up to three 5 ml spoonsful to be taken twice a day*	*one drop once daily l eye* *four times a day if needed* *one to be applied two times week* *two times every week when required*

We note that prescription directions do not contain information about the prescribed drug itself, nor do they typically specify mode and reason for it being prescribed; this information is however available in other structured attributes in the database and can be therefore easily retrieved. We also note that access to data from CPRD is subject to a full licence agreement and an Independent Scientific Advisory Committee approval.

In order to comprehensively represent the information in free-text medication prescriptions, we have designed and developed a model that records the following four attributes: dose number, dose frequency, dose interval and dose unit.

*Dose number* is the number of medication units taken in a single dose. We record the minimum and maximum dose number as prescribed, along with the *dose unit* (e.g., *capsule*, *tablet*), which represents the item of medication taken. If there is a choice left as to how much the patient can take, the values for the minimum and maximum will be different; otherwise these will be equal. For example, expression *“2-4 tablets”* is represented as a minimum dose of 2 and a maximum dose of 4, with “*tablet”* as the unit. In expression “*5 ml”,* both the minimum and maximum dose will be set to 5, with “*ml”* set as the unit. If there is an option not to take medication (e.g., “*up to 2 tablets”*), the minimum dose is set to 0.

*Dose frequency* represents the number of times the dose is taken in the dose interval – we use day as the default interval for the medication administration unless it has been stated otherwise (see below). For example, if a medication is taken every 4 h, then the frequency is 6 times per day. We record the minimum and maximum dose frequency. For example, in the expression “*3-4 times*”, the minimum frequency is 3 (times per day), and the maximum frequency is 4. If there is no choice, the value for both the minimum and maximum is the same. In cases where the prescription offers an option to take medication if/when required (e.g., “*take one every day when required”*), the minimum frequency is set to 0.

*Dose interval* is the time interval to which the dose frequency applies. For example, if a medication is taken daily, then the interval refers to the daily application. We use “daily” as the main (default) unit; if a medication is taken weekly, then the interval is 7 days. We record the minimum and maximum dose interval. For example, in the expression “*every 3 to 5 days*”, the minimum interval is 3 (days), and the maximum interval is 5. If there is no choice, the value for the minimum and maximum will be the same. For example, if a medication is taken on alternate days, the minimum and maximum dose intervals are set to 2 days between the administrations of the medication.

Table 1 provides examples of prescriptions with their respective representation in our model. In cases when certain information is not available (i.e. not expressed in the prescription), we use ‘?’ to record an unspecified value. Note, however, that in cases where the dose interval is missing, we assume that it is “daily” (unless the prescription refers to another source (e.g. *“as directed”*)).

**Table 1:** Examples of prescription instructions represented in our model

Prescription	dn_min	dn_max	df_min	df_max	di_min	di_max	dose unit
take 2 tablets 4 times a day	2	2	4	4	1	1	tablet
2 tabs qid	2	2	4	4	1	1	tablet
a half to one tablet to 2 three times a day when required	0.5	2	0	3	1	1	tablet
10 mg to be taken weekly	10	10	1	1	7	7	mg
2 with each meal	2	2	3	3	1	1	?
take 2.5 ml twice a day	2.5	2.5	2	2	1	1	ml
half a tablet twice a day when required	0.5	0.5	0	2	1	1	tablet
2 puffs 6 hrly prn	2	2	0	4	1	1	puff
1 to 3 every day	1	3	1	1	1	1	?
one or two to be taken every 4 to 6 hours	1	2	4	6	1	1	?
take as directed	1	?	?	?	1	?	-
apply as needed	1	1	0	?	1	?	-

*dn_min* is dose number (minimum), *dn_max* is dose number (maximum), *df_min* is dose frequency (minimum), *df_max* is dose frequency (maximum), *di_min* is dose interval (minimum), *di_max* is dose interval (maximum). Additional file 1: Table S1 contains examples of frequent Latin abbreviations

### Extraction of dosage information

Medication prescriptions recorded in rubrics are often dense with information, some of which can be ambiguous due to abbreviations, often of Latin nomenclature such as *“tds”* (Lat. *ter die sumendus,* three times per day, see Additional file 1: Table S1 for common examples), and confusing (numerical) lists of value (e.g., “*take 2 3 4 times per day*”). In addition, they often contain a number of typographical errors and misspellings.

**Fig. 1:**
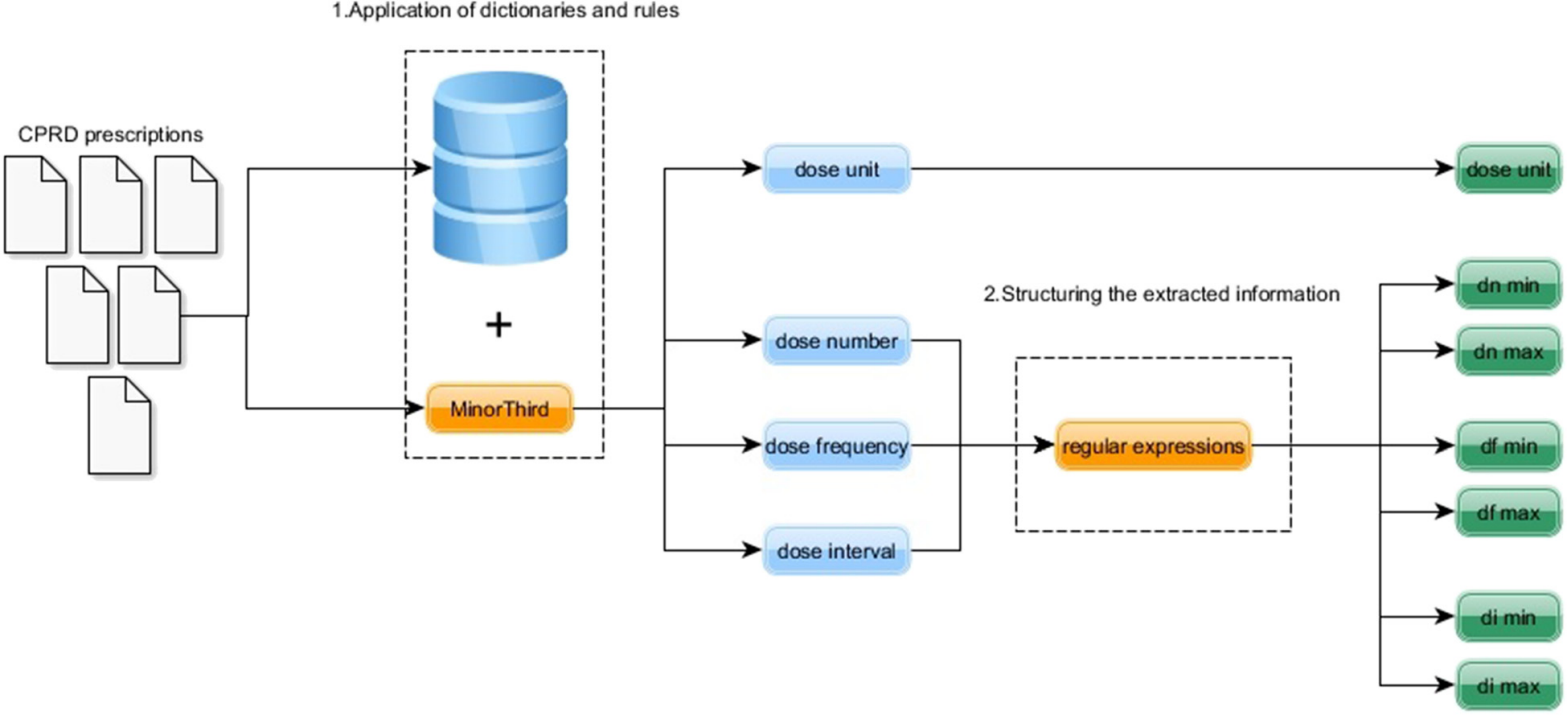
The two-step approach for the extraction of structured dose information from CPRD prescription instructions

A rule-based system was designed and implemented for the extraction of detailed prescription information. The approach has two steps (Fig. 1). In the first step, a set of dictionaries and rules are applied to the free-text prescription aiming to identify and populate candidate values in the model instance for that prescription. In the second step, a number of *meta*-*rules* are applied to provide consistency, set defaults for missing values or correct any values regarded as likely to be incorrect. These steps are explained below.

#### Step 1: Application of dictionaries and rules

A random sample of 200 free-text prescription instructions was chosen from more than 56,000 most commonly used CPRD instructions, and was manually reviewed in order to identify their lexical composition and engineer rules. A set of dictionaries (a total of 13, see Additional file 1: Table S2) was created for various medication attributes that appear in prescription free text, including common expressions for dose units, Latin abbreviations used for dose frequency, periods, etc. Around 300 generic rules were manually designed to model the three aspects of medication prescription: dose number, frequency and interval; we have also engineered rules to identify mentions of dose units. The rules rely on two types of constituent:

- specific semantic classes (e.g., expressions referring to meals or numbers); these are either represented by the dictionaries or modelled using regular expressions (i.e. patterns that match character combinations in text strings; e.g., standard numerical expressions are represented as series of digits). These classes can include lexical variation: for example, numbers (*NUM*) can include not only standard numerical expressions, but numerical ranges (e.g., *2-3*) or numerals expressed in words (e.g., *“two”*).
- semi-frozen lexical expressions, used as anchors or context for mentions of certain types of medication information. For example, we modelled a variety of lexico-syntactic expressions that indicate expressions for administration of a medication such as “*take* NUM *pills*”, where *NUM* indicates a number or range (as above).

The rules are designed as syntactic patterns consisting of these two constituents (i.e. semi-frozen chunks and/or semantic place holders). For example, a rule that captures the minimum and maximum dose number would have two parts: a semi frozen verbal expression (e.g., “*take*”, “*inhale*”, “*use*”), followed by a numeric mention matched by the numeric regular expressions and a possible time unit (“*2 a day*”, “*3-4” every week*”). Table 2 shows the number of rules created for each of the medication prescription attributes (dose, frequency, interval, unit), along with some typical examples. For the implementation of the rules we used MinorThird [29], an information extraction development environment, with default, built-in tokenisation.

**Table 2:**
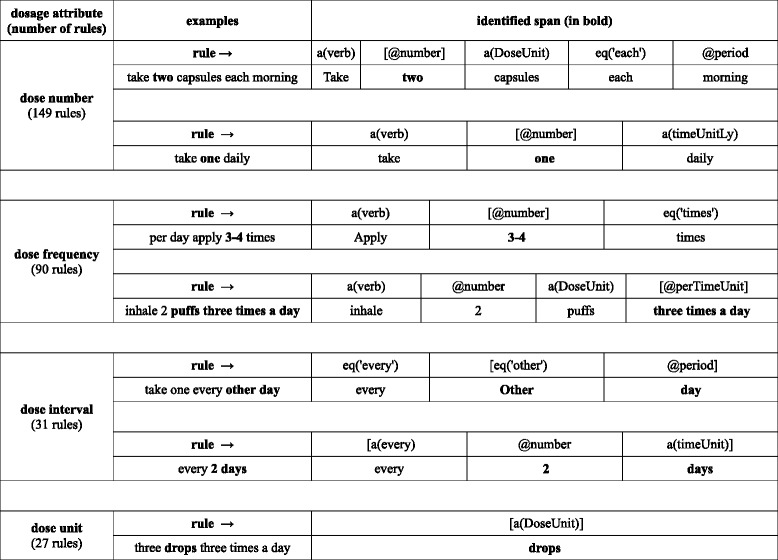
Examples of rules for the recognition of dosage attributes in medication data

The rules were implemented in MinorThird [29] and we use its notation here. Only the part in brackets (the string of interest) is being extracted as a mention (i.e., annotation); the rest of the rule (if any) specifies the context/anchors. The rules use explicit matching of spans (e.g., *eq(‘times’)*), the dictionary matches for single (e.g. *a(verb)* – matching verbs that indicate the administration of a medication (e.g., *take, insert*)) and multiword terms (e.g., *@period*, see Additional file 1: Table S2). *Number* models numerical expressions including those belonging to the dictionary “*number*”; a(timeUnitLy) matches the words of the dictionary “*timeUnitLy*” with prescription text that indicates an adverb of time e.g., “*daily*”, “*weekly*”, etc; @perTimeUnit recognises syntactical patterns of the dictionary “*perTimeUnit*” that contain both numeric and word phrases in prescription text e.g., “*four times a day*”, “*2 times per week*”, etc.; a(timeUnit) identifies the words from the “*timeUnit*” dictionary (see Additional file 1: Table S2)

#### Step 2: Structuring the extracted information

The rules applied in Step 1 aim to identify candidate mentions that correspond to a specific attribute. The recognised candidate mentions are then used to capture the minimum and maximum values of each attribute using a number of rules that capture lexical expressions of ranges (e.g., “2 to 4”, “2-4”, “between 2 and 4”). Additional rules have been created to deal with optional values (e.g., *“up to NUM”*, or matching specific expressions such as “*as necessary*”, “*when required*”, “*if needed*”); in such cases, the minimum dose frequency is set to 0. A number of defaults were used in cases where information is not explicitly represented in the prescription text. For example,

- The minimum and maximum dose numbers are set to “1” in cases where the prescription text is not specific about dose numbers, but still contains information for the identification of dose frequency (e.g., the dose number is set to 1 for all of these “*apply four times a day when required*”, “*to each eye every four hours”*, “*every morning after food*”, “*take in the morning for blood pressure*”).
- The minimum and the maximum dose number are set as an average in prescriptions that include two (or more) unequal doses per day (e.g. the dose number in *“one every morning and two every night”* is set to 1.5). We record the dosage frequency in the standard way (“2” in the previous example, as the medication is to be taken twice (morning, night)), so that the total daily dosage can be inferred (see also discussion in Section 3.3).
- The minimum dose interval is set to “1” and the maximum to “*?*” when the prescription text contains no information that suggests any specific detail or context (e.g., *“as directed”*).
- Common general meal-time expressions (e.g., *“with each meal”* or *“after meal”*) were assigned a daily frequency of 3, with individual meals counted as frequency of 1 (e.g., “*with breakfast*”).

This step is also used to check consistency (i.e. that the minimum value is not bigger than the maximum), and inconsistent cases would be discarded.

## Results and discussions

We performed two experiments: first, using a small gold-standard sample, we aimed to evaluate the performance of the proposed text mining approach (Section 3.1). Then, using a larger set, we aimed to explore, quantify and discuss the practice in medication prescriptions from CPRD (Section 3.2). The study was approved by the CPRD Independent Scientific Advisory Committee (ref 11_154A).

### Gold-standard evaluation

The initial set of 200 free-text CPRD prescription instructions was used to develop and tune the system, and these were not used for the evaluation. Following the development of the system and in order to create a gold standard for the evaluation, a new set of 100 medication prescriptions was randomly selected from the CPRD dataset of 56,000 free-text prescriptions, and manually and independently annotated by the authors: a clinical consultant (WGD), a health informatician (GN) and a health informatician with medical background (GK). The initial inter-annotator agreement was 93 % at the prescription level, calculated by the absolute agreement rate [30], indicating a very good annotation consistency. All disagreement cases were reviewed, and were considered omissions (e.g., a wrongly recorded maximum dose number) rather than fundamental disagreement. The data were then corrected by the agreement of all annotators. Following this, another random set of 120 CPRD prescriptions was manually annotated by GK and merged with the 100 gold-standard prescription instructions to form the final evaluation dataset.

Out of the 220 prescription instructions in the gold-standard dataset, 33 (15 %) contained different values for minimum/maximum values in at least one of the attributes, and a further 26 (12 %) contained at least one of the minimum/maximum values unspecified (i.e. represented as ‘?’).

The prescription extraction system was then applied to the gold-standard set and the results were compared to the human annotations. We used accuracy to assess the system’s performance, defined as the number of correctly identified and structured medication attributes (true positives) divided by the total number of prescription attributes in the evaluation set. We calculated the accuracy at both the dosage attribute levels and also at the prescription level.

Table 3 provides the detailed results at the dosage attribute level. The accuracy values were ranging from 94 %-100 %, suggesting reliable results across all the medication attributes. The minimum dose interval and dose unit had the highest accuracy (100 %), while maximum dose frequency had the lowest accuracy of 94 %. At the prescription level, each free text record is considered a true positive only if all its attributes have been successfully identified. The accuracy at the prescription level was 90.9 %, suggesting relatively reliable results.

**Table 3:** The accuracy of the medication attribute extraction

Dosage attribute	True positives (out of 220)	Accuracy (%)
dose number (minimum)	211	95.9
dose number (maximum)	210	95.4
dose frequency (minimum)	210	95.4
dose frequency (maximum)	207	94.0
dose interval (minimum)	220	100.0
dose interval (maximum)	217	98.6
dose unit	220	100.0
(macro) accuracy	97.0	

Accuracy is shown for each attribute, considered separately. Macro accuracy represents an average of the accuracy values across different attributes

By analysing the results, two bugs were identified caused by oversights in the system implementation: while we handle half dose units generally, due to a hiccup it was not included in the rule for handling cases of varying administration at different times (e.g. in “*one in the morning and half at night*”). Another oversight was missing “or” as a range operator for the expression of alternative dosages (e.g. in *“20 or 40 mg before meals four times a day”*). We note that these bugs were corrected before using the system for the analysis described below.

### Analysis of free-text instructions in CPRD

We applied the system to 56,114 most common free text instructions within CPRD (see next paragraph), to explore how much variability (i.e. minimum, maximum values, optional dosages) is presented in free text prescriptions. We note that here we do not aim to look at the variability of prescriptions across and between patients, but rather the level of flexibility in a single prescription.

The data set was obtained from the “common_dosages” table in CPRD, which is a generic look-up table of most common free text instructions that GPs type within electronic prescriptions. This table is a generic repository of all different (unique) free-text directions that have been collected from the whole CPRD for all treatments and diseases. Each row corresponds with instructions about how a drug is to be taken (but no information about the drug itself is included – this information is available in other tables).

We note that for a total of 406 records (0.72 %), we were not able to extract any dosage information. A manual review of a random sample of 30 such prescriptions showed that indeed no useful information was present that could be extracted (e.g., “~ ~ ~ ~ ~ ~ ~”, “*1-2 four*”, “*28percent*”, “*40n*”, “*human*”). We also note that there were no cases that were discarded because of inconsistent values (i.e. the minimum value is bigger than the maximum).

Table 4 provides an overview of the dosage attributes identified in this data set. Almost a quarter of prescriptions (24 %) have variability in at least one attribute (i.e. different min/max value, where both values are specific): 11 % had different minimum and maximum dose numbers (e.g., *“2-4 tablets”*) and 18 % had different minimum and maximum dose frequencies (e.g., “*2-3 times”*). Only 55 examples (less than 0.1 %) had different minimum and maximum dose intervals (e.g., *“every 2-3 months”*).

**Table 4:** Medication prescription variability in the most common CPRD prescription instructions

Prescriptions with	Number of such prescriptions (out of 56,114)	Prescriptions percentage
all medication elements as “?”	406	0.7 %
at least one element as “?”	11,696	20.8 %
dn_min ≠ dn_max	6,278	11.1 %
df_min ≠ df_max	10,249	18.2 %
di_min ≠ di_max	55	0.1 %
no dose units	36,111	65.4 %

*dn_min* is dose number (minimum), *dn_max* is dose number (maximum), *df_min* is dose frequency (minimum), *df_max* is dose frequency (maximum), *di_min* is dose interval (minimum), *di_max* is dose interval (maximum)

A fifth of prescriptions (20 %) had at least one unspecified dosage attribute (either minimum or maximum dose number, frequency or interval). We note that there were no unspecified values in dose numbers as we have provided defaults when these are not explicit. On the other hand, almost two thirds of the prescription instructions did not contain information regarding dose units, but this information is available from other tables within CPRD. Still, a total of 22 different dose units were recognised in free-text instructions, with *tablet* and *millilitres* as most frequent dose units (30 % and 27.5 % respectively).

### Discussion

Our analysis of free-text prescriptions in the CPRD database shows that a substantial proportion of records have flexibility or variability in prescribed dosage and/or frequency, and therefore the ability to represent such details is key for supporting pharmacoepidemiology researchers in preparing prescription data for further processing. We note that the CPRD database does contain transformation of the free text prescriptions into various structured dose variables, but their model does not allow for further choices that the researchers can make in cases where there is variability or flexibility in drug administration, as in such cases the attributes are recorded by a single average value, without specifying prescribed options or ranges. In particular, cases where drugs are taken *when* or *if needed* are not recorded. Our model, on the other hand, allows researchers to explore effects of all such variability by making transparent decisions as to which values have been taken into account.

While the quality of text-mined data was high, there were still cases where the system failed to extract correct information. Such cases in the evaluation gold-standard dataset were analysed in detail (see Additional file 1: Table S3 for all errors). We summarise here the major challenges:

- Misspellings: As with other types of clinical text [31, 32], prescription records often contain misspellings (e.g., “*2bd bil*””, “*aplly twice daily*”). For example, in our evaluation set, 11.8 % (26 out 220) of prescription instructions had misspellings. A common source of errors is the misspelling of dose units or frequency keywords, which have failed to trigger relevant rules. In such cases, most if not all of dose attributes would be unrecognised (not only the one linked to the cue word in question). For example, half of the cases where the dose number was incorrectly identified were due to misspellings (e.g. “*1 mnae*”, “*2-3 spoonsfuls to be taken twice daily*”), as the system failed to recognise misspelled keywords (e.g., “*mnae*” for “*mane*”; “*spoonsfuls*” for *“spoonfuls”*), which are used to trigger the associated rules.
- Tokenisation: The tokenisation approach used in MinorThird has resulted in a number of errors. For example, our pre-processor did not tokenise on ‘/’ (e.g., “*take 1 mane/take 1 at night*” has “*mane/take”* as a single token). In addition, spaces were sometimes missing between dosage attributes (e.g., dose number and dose frequency - “*1mnae*”, “*1-26hlryprn*”), which makes it challenging to identify correct tokens.
- Structural ambiguity: There are cases where the prescription text can be interpreted in more than one way, often as a result of typographic errors or omissions. For example, prescription “*take 1 2 3 times per day*” can be read as “*take [1 or 2 DOSE UNITS], [3 times per day]*” or “*take [1 DOSE UNIT], [2-3 times per day]*”. A similar case is *“a half to one tablet to 2 three times a day when required”*, which can be either interpreted as *“[a half to one tablet] [to 2 three times] a day [when required]”* (“0.5 to 1 tablets, 2-3 times a day when required”) or *“[a half to one tablet to 2] [three times] a day [when required]”* (“0.5 to 2 tablets, 3 times a day when required”). Our system opted for the latter, identifying the second *“to”* as a dosage number cue rather than a frequency cue (and not realising that it is likely that there was a typo where “to” and “2” were swapped in the original text). Similarly, other types of ambiguity also proved challenging. For example, prescription *“6 per day”* can mean either 6 dose units taken at once, or one taken 6 times. While being ambiguous, we note that the pattern of administration within a day may not be important for a given data analysis (and thus may not matter which one – in this case – is extracted). Another example is “*2 for pain*”, where the missing interval (per day or just one-off) makes the whole expression under-specified. Another example is “*apply 2 times a day when required*”, which could be interpreted as “twice a day on days when required” or “apply daily up to 2 times when required”.
- Lexical coverage in the dictionaries: Despite a reasonable assumption that the prescription lexical space is limited, we have still encountered cases where the dictionaries were not complete in particular for some variation of common concepts (e.g., “tablet(s)”).
- Acronyms and abbreviations: Medical abbreviations are used extensively, and they appear in multiple ways. Acronyms can also be ambiguous (e.g., “*od*” that can stand either for “*once a day*” or for “oculus dexter”, *right eye*), and additional information (e.g., other linked tables in CPRD) may need to be used to disambiguate them.
- Varying dosages: While we aimed to model (and extract) cases where there were different doses taken in different times (e.g., *“one every morning and two every night”*, “*take 3 in the morning 2 at teattime and 3 at night*”), the system missed some specific patterns of variability. For example, there were cases where prescriptions require administration at different specific time(s) on the hour scale (e.g., “*take one tablet and then at 8 am and one at 2 pm*”, “*1 at 8 am 1 at 4 am*”). We note that the model would need to be expanded to allow representation of specific dosages for each administration point in cases where such granularity is of interest for drug exposure modelling (e.g., “*take 3 in the morning 2 at teattime and 3 at night*” can be modelled as three parallel drug administration events – one for morning, one for mid-day and one for night).
- Drug administration duration and breaks: A special type of prescription is that which asks for a specific duration or requires breaks in taking medication (e.g., “*1 daily for 21 days then 7 day break caution if vomiting diarrhoea antibiotics*”, “*2 drops to each eye* every *2 hour for 24 hour then 2 four times a day til settled for 2 day*”; “*2 to start then 1 after each loose motion up to 16 mg total daily*”). This type of prescription instruction requires an extended model to represent the dynamics of the drug administration. There are also examples that suggest a single, one off, dosage administration (e.g., “*take 1 at 9 o clock*”), which is not currently supported by our model.

Our rule-based approach to the extraction of prescription details proved to be both effective and efficient. While building rule-based systems is often time consuming, in this case, the whole system was engineered within two months of effort, requiring further two months for tuning and adjustments. The complementarity of expertise within the development team covered both clinical aspects and text mining experience, which led to the rapid implementation of the lexicalised rules. Given the modular implementation, any further changes in the model can be relatively quickly deployed.

## Conclusion

Electronic health record research databases, such as the CPRD, contain a wealth of patient information including coded data and information that appears only in unstructured text. This includes free-text directions from the prescribing doctor associated with medication prescriptions. In this paper we introduced a model and presented a rule-based system for the identification of detailed structured medication dosage attributes (dose number, dose frequency, dose interval, dose unit). The system specifically captures the variability and flexibility in instructions, such as different minimum and maximum dosages. The evaluation process revealed reliable performance with an overall accuracy of over 90 %, suggesting that the proposed implementation can be useful for exploring prescription patterns on a large scale. In the analysis of most common free-text prescriptions in the CPRD database, we were able to demonstrate that at least a quarter of prescriptions have some level of variability or flexibility. By capturing the variability in the possible range of exposures from a single prescription, we allow the researcher to select how they would like to model the exposure, for example selecting the minimum dose number, maximum dose number, average dose number or a random dose number within the minimum to maximum range. While some issues still remain for future work (e.g. handling dosage duration, varying dosages and breaks/different dynamics in medication administration), the current model can be used to prepare drug exposure information for epidemiological studies.

### Availability of the resources

The software and evaluation data is available at http://gnteam.cs.manchester.ac.uk/resources/DOSES/.

## Additional file

Additional file 1:
**Table S1.** Examples of commonly used Latin abbreviations in medication. **Table S2.** Dictionaries used for the identification of dosage information. **Table S3.** System errors from the test dataset (220 prescriptions). (DOCX 25 kb)

## Abbreviations

CPRD: Clinical Practice Research Datalink
EHR: Electronic health record
CRF: Conditional random field
SVM: Support vector machine
MedXN: Medication extraction and normalization system
dn_min: Minimum dose number
dn_max: Maximum dose number
df_min: Minimum dose frequency
df_max: Maximum dose frequency
di_min: Minimum dose interval
di_max: Maximum dose interval
