# Supplementary material for: Modelling and extraction of variability in free-text medication prescriptions from an anonymised primary care electronic medical record research database
Source: BMC Med Inform Decis Mak. 2016 Feb 9;16:18. doi: 10.1186/s12911-016-0255-x (PMC4748480; doi:10.1186/s12911-016-0255-x)
Supplement: Additional file 1: — Table S1. Examples of commonly used Latin abbreviations in medication. Table S2. Dictionaries used for the identification of dosage information. Table S3. System errors from the test dataset (220 prescriptions). (DOCX 25 kb) [file 12911_2016_255_MOESM1_ESM.docx]

**Modelling and Extraction of Detailed Medication Prescription
Information from an Anonymised Primary Care Electronic Medical Record Research Database**

**Supplementary materials**

| **Latin abbreviation** | **Full form** | **Meaning** |
| --- | --- | --- |
| bd (s), bis, b.i.d. b.d. | bis in die (sumendum) | twice daily |
| b.t, h.s | bedtime, hora somni | bedtime |
| dieb alt  eod | diebus alternis  every other day | every other day |
| nocte, noct | at night | at night |
| od  opd, o.p.d  sid, s.i.d | omne in die  once per day  semel in die | once per day |
| om | omne mane | every morning |
| on | omne nocte | every night |
| qad, q.a.d, | quaque alternis die | every other day |
| qd, q.d | quaque die | every day |
| qds, qid | quaque die sumndus, quarter in die | four times a day |
| tds, t.d.s | ter in die | three time a day |

**Table A1:** Examples of commonly used Latin abbreviations in medication.

| **Dictionary (size)** | **Description** | **Example** |
| --- | --- | --- |
| dose units (73) | dose units in which a medication is being administered. | “*pills*”, “*drops*” |
| numbers (20) | numerical vocabulary that includes numbers and words that express numbers. | “*1*”, “*twenty* *two*” |
| administration verbs (20) | verbs that indicate the administration/taking of a dose. | “*take*”, “*inject*” |
| periods (27) | English and Latin words for periods that a medication dose is being administered. | “*morning*”, “*nocte*”, “*evening*” |
| adverbial time units (19) | adverbs indicating the interval of the dose administration. | “*hourly*”, “*weekly*” |
| nominal time unit (18) | nouns that display temporal information regarding the administration of a dose. | “*minutes*”, “*hours*” |
| temporal abbreviations (10) | abbreviations for time periods for the administration of medications. | “*pm*”, “*am*” |
| alternate (5) | words for the administration of medication dosage in alternate time periods | “*alternate*”, “*dieb* *alt*” |
| Latin abbreviations (108) | Latin abbreviations used in medication prescriptions to indicate dose frequency (see also Table 3). | “*tid*”, “*bd*”, “*bis*” |
| Roman letters (10) | Roman letters from one to 10. | “*I*”, “*III*” |
| meals (9) | words representing meals. | “*meal*”, “*supper*” |
| temporal links (6) | prepositions suggesting a medication administration in relation to time. | “*before*”, “*after*” |
| day of the week (14) | days of the week including shorthanded textual representations | “*Monday*”, “*Tuesd*” |

**Table A2:** Dictionaries used for the identification of dosage information.

The dictionaries contain synonyms and other variations (e.g., Latin medical abbreviations for “*bd*” include “*b.d*.”, “*bis*”, “*b.i.s*”, “*bds*”, “*b.d.s*”, etc.), hence the relatively high size for some dictionaries.

| **Prescription** | **dn_min** | **dn_max** | **df_min** | **df_max** | **di_min** | **di_max** | **dose_unit** |
| --- | --- | --- | --- | --- | --- | --- | --- |
| *alternate mornings* | 1 | 1 | **1** [?] | **1** [?] | 2 | 2 | - |
| *one in morning and half at night* | **1** [0.75] | **1** [0.75] | **1** [2] | **1** [2] | 1 | 1 | - |
| *2 drops to each eye every 2 hour for 24 hour then 2 four times a day til settled for 2 day* | 2 | 2 | **4** [?] | **4** [?] | 1 | 1 | drop |
| *6 per day* | 6 [1/?] | 6 [?] | **6** [1/?] | **6** [?] | 1 | 1 | - |
| *2 to start then 1 after each loose motion up to 16mg total daily* | **2** [1] | **2** [?] | 1 | 1 [?] | 1 | 1 | mg |
| *1 at 8am 1 at 4pm* | **4.5** [1] | **4.5** [1] | **1** [2] | **1** [2] | 1 | 1 | - |
| *2-3 spoonsfuls to be taken twice daily* | **1** [2] | **1** [3] | 2 | 2 | 1 | 1 | - |
| *1-26hlryprn* | **?** [1] | **?** [26] | 0 | **?** [1] | 1 | ? | - |
| *2 for pain* | **?** [2] | **?** [2] | ? | ? | 1 | ? | - |
| *take 1 mane/1 at night* | 1 | 1 | **1** [2] | **1** [2] | 1 | 1 | - |
| *half tablet(s) or one tab alternate days* | 0.5 | **0.5** [1] | 1 | 1 | 2 | 2 | [tab] |
| *take 1 at 8 o clock* | 1 | 1 | **?** [1] | **?** [1] | 1 | ? [1] | - |
| *up to three 5ml spoonsful to be taken twice a day* | **15** [5] | 15 | 2 | 2 | 1 | 1 | ml |
| *two times every week when required* | ? | ? | 0 | **1** [2] | 7 | 7 | - |
| *20 or 40mg before meals four times a day* | 20 | **20** [40] | 4 | 4 | 1 | 1 | mg |
| *1marne* | **?** [1] | **?** [1] | **?** [1] | **?** [1] | 1 | **?** [1] | - |
| *1 tablet(s) with breakfast* | 1 | 1 | **?** [1] | **?** [1] | 1 | ? [1] | [tab] |
| *1mnae* | **?** [1] | **?** [1] | **?** [1] | **?** [1] | 1 | **?** [1] | - |
| *10 ml in bath if required* | 10 | 10 | 0 | **?** [1] | 1 | ? | ml |

**Table A.3:** System errors from the test dataset (220 prescriptions)
Highlighted in bold is the value returned by the system, while the correct value is given in [ ].
